# Addressing heterogeneity in the development of circular economy strategies in the offshore wind industry: A review

**DOI:** 10.1016/j.heliyon.2024.e39577

**Published:** 2024-10-22

**Authors:** Pankaj Ravindra Gode, Arild Aspelund

**Affiliations:** Norwegian University of Science and Technology (NTNU) Department for Industrial Economics and Technology Management, Sentralbygg 2, Trondheim, Norway

**Keywords:** Circular economy, Offshore wind industry, Waste management, Technology readiness level, System-level change

## Abstract

To mitigate the impact of climate change, a transition to renewables in the energy sector is needed. Offshore wind energy is a promising renewable technology that is developing at an unprecedented rate. However, the offshore wind industry is material intensive, and there are concerns about its end-of-life strategies and that we might end up exchanging a climate challenge with a material challenge. The question is whether there are available, mature, and commercially viable solutions for offshore wind companies that want to adopt a circular economy. This study offers an exhaustive review of the academic literature on eight existing and emerging circular economy strategies for the offshore wind industry – lifetime extension, reuse, remanufacturing, refurbishing, repurposing, modularisation, repowering and recycling, and classifying them according to the technology readiness level**/**strategy assessment framework. The systematic literature review identified 66 relevant articles using two databases and a keyword search. The main conclusion is that the circular economy in the offshore wind industry is an under-researched, underdeveloped field with great heterogeneity in the development of circular economy strategies. This heterogeneity is caused by wide variation in the country and region-specific development of circular economy strategies, policy and regulatory factors, economic viability, and lack of knowledge. We also find that the circular economy strategies with potentially the best environmental outcome are the ones that are furthest from market maturity and commercial viability.

## Highlights

• Large-scale decommissioning of offshore wind farms will begin in the 2030s.
• Circular economy is gaining traction in the offshore wind industry to manage waste.
• Recycling is the most developed CE strategy in the offshore wind industry.
• CE strategies with higher sustainability potential are underdeveloped.
• System-level change is needed to develop CE strategies further.

## Graphical abstract

**Figure:**
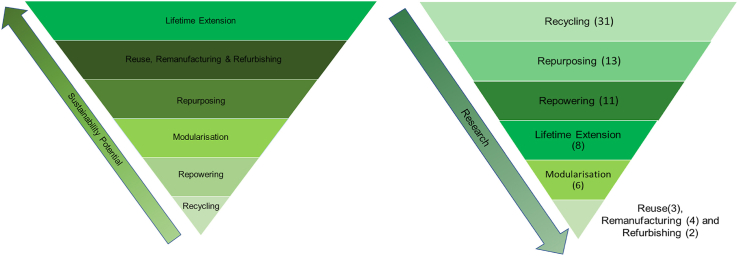

## 1 Introduction

**Fig. 1:**
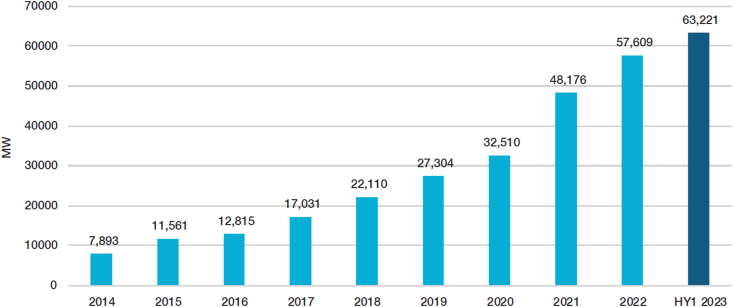
Global offshore wind capacity in operation – cumulative [3].

To address climate change impacts and limit the global average temperature rise to 1.5 °C above pre-industrial levels, significant changes in all facets of society are necessary [1]. Accelerating the deployment of renewables will be one such pathway that could aid rapid decarbonisation of the current energy mix [1]. Offshore wind (OW) is one of the renewable energy sources that is currently developing at an unprecedented rate. According to the World Forum of Offshore Wind, around 63,000 MW of offshore wind capacity was in operation in the first half of 2023 (Fig. 1), and this figure is expected to increase to 447,000 MW by the end of 2032 [2,3]. Moreover, the offshore wind turbine size is expected to grow even further – there has been a 16 % increase in turbine capacity per year [4], with China's Three Georges Corporation currently operating the world's first 16 MW turbine at Zhangpu Liuao offshore wind farm [5]. With regard to such exponential growth, there have been concerns about end-of-life (EOL) strategies of anthropogenic material [6], as some of the first-generation offshore wind farms will be reaching the end of their expected lifespan by the 2030s [7]. There are estimates that by 2035, over 3500 MW of global offshore wind capacity will have reached the end of its life [8]. Currently, decommissioning plans for offshore wind farms rarely include detailed information on EOL management for the wind farm infrastructure [[8], [9], [10]]. This is mainly due to the limited experience in carrying out the decommissioning of offshore wind farms, as only a few offshore wind farms have been decommissioned to date [9]. This lack of experience in carrying out decommissioning activities leads to uncertainties in EOL planning and results in significant waste production and environmental impacts [11]. Cumulatively, it explains why the OW industry needs to look at sustainable ways of managing waste. Currently, such sustainable waste management pathways that the OW industry is considering fall under the umbrella term of circular economy (CE) [12].

As we will see, there are several strategies to attain circularity. In the case of the OW industry, Jensen et al. (2020) argue that emphasis should be on component durability, longevity, impact recovery, direct reuse, and, whenever required, recycling and remanufacturing [9]. Arguably, there are several reasons why the CE is well-suited for the OW industry. Embedding a CE in the OW industry will not only minimise the extent of resources used and maximise the prevention of waste but will also offer a range of economic, environmental, social and technical benefits [10,13]. For example, it can create new revenue streams by presenting new market opportunities for recovered products [14] – such as the use of wind turbine blades to make bridges, furniture and playground equipment, and the use of foundations and seabed structures to make artificial reefs [11,[15], [16], [17], [18], [19]]. It can yield significant cost savings. For example, a case study conducted on a London wind farm showed that extending the lifespan of the existing wind farm or retrofitting turbines with higher capacity could result in capital savings of up to £2 billion [20]. Another study reported that repurposing 20 % of Irish blade waste for second-life application would avoid 135 tonnes of blade waste ending up in landfill and would displace 30,780 kg of CO_2_ emissions annually [21]. Introducing circular business models in the wind industry would also lead to job creation through market diversification, local community development, education and upskilling, and the development of renewable energy solutions for developing economies [22].

Although the potential gains are significant, there are challenges to the successful implementation of CE strategies in the OW industry. Recent research has shown that it is crucial to gather data on different cycle stages of the wind farm, along with necessary technical, economic and social information [22,23]. Moreover, strong collaboration between industry, research and governmental organisations is also required [[24], [25], [26]]. There are several such promising collaborations taking place throughout the offshore wind industry. Examples include the GENVIND project (2012–2016), which investigated ways of reusing composite waste [27,28], the Re-Wind project (2018–2021), which investigated repurposing wind turbine blades to make structural components [21,24], and the DecomBlades project (2021–2023), which developed Blade Material Passports to support the commercial development of recycling technologies [29,30]. For successful implementation of a CE, it is also necessary for such collaborations to have relevant regulatory support, standardisation and reliable economic analysis [22,26]. To complicate matters further, there are also idiosyncrasies to every offshore wind farm that lead to EOL solutions being highly site-specific [20,[31], [32], [33]].

There is, however, an even more fundamental factor that might explain the limited adoption of CE practices in OW industry. According to recent research, there seems to be great variation in the development (and hence commercial availability and feasibility) of CE strategies, such as lifetime extension, reuse, remanufacturing, refurbishing, repurposing, modularisation, repowering and recycling in OW [9,12]. This heterogeneity can help to provide an understanding of actual CE adoption in OW industry.

This study seeks to contribute to this discussion by investigating the following research question (RQ): what is the state-of-the-art in availability, heterogeneity and development of circular economy strategies in the OW industry? The unique contribution of this study comes from reviewing the technological and market maturity of current and emerging CE strategies for the OW industry, discussing the reasons behind the heterogeneity of their development, and suggesting systematic changes that are needed in the assessment of CE strategies to accelerate the CE transition in the OW industry. The focus of the article is on the offshore wind industry because of its higher social acceptance and anticipated levelised cost of energy (LCOE) reduction, its predicted share in the renewable energy mix, and the exponential growth of turbine sizes that causes EOL management to be a significant challenge [12].

The paper is structured as follows. The next section provides an industry outlook and defines CE strategies according to their sustainability potential. This is followed by a review of the methodology, which describes the manners in which the literature has been identified and evaluated using the technology readiness level (TRL) group/strategy assessment framework. Finally, the findings are presented, analysed and discussed according to the RQ presented above.

## 2 Industry outlook and circular economy concepts

In order to address the RQ, it is necessary to contextualise the OW industry, discuss relevant policy & regulatory support, and define the relevant CE strategies and how they relate to each other.

### 2.1 Current industry outlook and necessary policy development

During the early years of offshore wind energy development, the industry was focused on increasing its overall deployment and reducing the LCOE. EOL strategies were given low priority and left for long-term consideration [8]. In more recent years, some OW farms were dismantled, but the experiences gained during the decommissioning of these wind farms are not openly available and have not been considered while developing new decommissioning plans [9]. Recent research suggests that new offshore wind farms in the UK lack information on material recovery rates, and decommissioning plans show little development [9].

One reason for the slow development of EOL strategies in the wind industry can be ascribed to costs. There are currently developed few, if any, EOL technologies that can compete with the low cost of energy recovery (incineration – combustion of organic parts of waste) and landfilling [[34], [35], [36], [37]]. However, due to the significant adverse environmental impacts of energy recovery and landfilling, countries and regions are implementing regulations against their usage [17,34,37,38]. This provides motivation for the OW industry to accelerate the transition to CE practices in decommissioning.

Another motivation is the growth of the OW sector. Currently, most windfarms that are approaching EOL are situated on or nearshore; however, the wind industry is increasingly moving offshore, which will result in higher material footprints as offshore windfarms are increasing in capacity and turbine sizes and need more specialised installation, maintenance, decommissioning vessels, and so on. Therefore, the growing material footprint of a rapidly growing industry requires attention.

To accelerate the CE transition, however, and to form a business case for CE strategies, it is vital to have strong and relevant policy support together with necessary incentives [26,39]. Such policies and regulations should also aim to improve localised infrastructure for handling and processing waste, strengthen research and innovation, and source locally available materials, where possible. A good example of such an initiative is the European Critical Raw Materials Act, which aims to strengthen and diversify Europe's critical raw materials supply and to develop the necessary infrastructure to avoid supply chain shortages that can be caused due to geopolitical tensions [40]. When promoting CE in the OW industry, policymakers can also emphasise the importance of resource efficiency on industrial actors, which can be achieved by minimising waste generation, ensuring efficient design, and preventive and scheduled maintenance [23,41]. Policymakers can also aid the development of extended producer responsibility (EPR) initiatives, where original equipment manufacturers (OEM) have to accept responsibility for their products in the downstream stages of recovery and EOL processing [6,23,32,41]. Enforcing EPR compliance with incentives or penalties can help to accelerate the transition to a CE [41]. The use of recycled and sustainable material in the construction of wind turbine components should also be promoted by policymakers [12,41].

### 2.2 The concept of the circular economy

The circular economy is an emerging research avenue with a wide range of definitions; in fact, a recent study showed the existence of 221 definitions for CE [42]. Whilst this is not entirely undesirable, due to the discursive nature of sustainability [43,44], particular care needs to be taken that such definitions show a clear relationship to sustainability, and that they are not only associated with specific CE strategies, such as reuse and recycling [42,45,46]. A single consensual definition of CE with a clear link to sustainable development would provide an excellent backbone for policymaking, as it would provide a rigid background, assumptions and targets around which policymaking can be formulated [42].

For the purpose of this article, we are adopting the following definition of the circular economy: “an economic system in which resource input and waste, emission, and energy leakages are minimized by cycling, extending, intensifying, and dematerializing material and energy loops” [[47] p. 3]. We have chosen this particular definition as we believe it goes beyond mainstream CE strategies of reuse and recycling, as mentioned above, and also provides a clear link to sustainability.

**Fig. 2:**
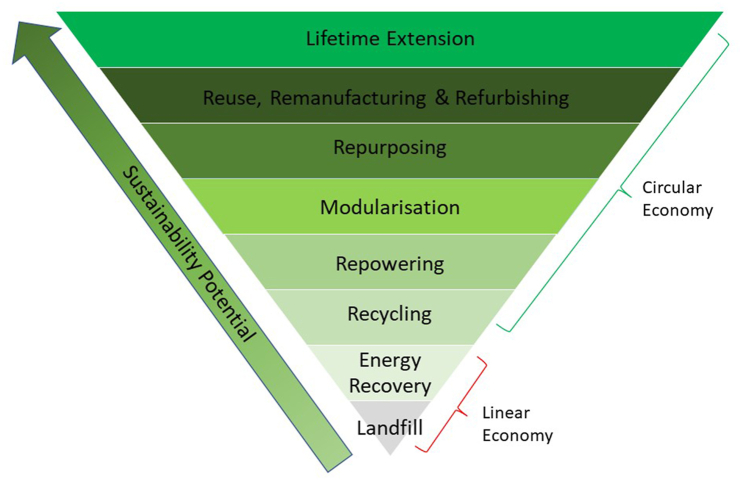
Linear & circular end-of-life strategy hierarchy for the wind industry according to their sustainability potential (adapted from Refs. [9,12,48])

Fig. 2 represents the hierarchy of waste management approaches in the wind industry [9,48]. The strategies shown in the pyramid are ranked according to their sustainability potential, where the waste management strategies highest in the hierarchy are the most desired from an environmental perspective [9]. The bottom two levels of the pyramid are part of the linear economy, and the top six levels represent the circular strategies. Current reviews of decommissioning plans for offshore wind farms suggest that, despite the awareness on the part of OEMs and wind farm operators, the waste management is currently dominated by the bottom three levels of the pyramid – i.e., recycling, energy recovery and landfill [9]. Whereas, despite their promising economic (e.g., raw material and waste reduction), environmental (e.g., carbon and energy saving) and social (e.g., job creation and skills development) contributions, the top five levels of the hierarchy (hereafter referred to as ‘higher-level CE strategies’) are found to be underdeveloped [9,46]. Apart from the already discussed economic factors, the literature also points out the need for innovation to develop these higher-level CE strategies [9].

### 2.3 Circular economy strategies used in the wind industry

In this section, we present the circular strategies defined by the waste management hierarchy and describe their application in the OW industry.

#### 2.3.1 Lifetime extension

Lifetime extension involves extending the service life of wind farm components beyond their designated lifespan [12]. In terms of environmental sustainability, lifetime extension is considered the most desirable strategy [9], as it can potentially reduce waste flows and resource use significantly [36,49]. This can be achieved by censoring, monitoring and systematic maintenance. In order to be most efficient, however, it needs to be integrated into the initial design phase of the assets to ensure possibilities for modularity and upgrading [49].

Despite the significant environmental benefits, the economic benefits of lifetime extension have been identified as a key hurdle. On the positive side, lifetime extension also extends the capital investments over a longer period and effectively increases operation time, energy generation and return on investment, which also reduces LCOE. It also increases operational costs due to increased maintenance, affecting lifetime extension's economic viability [50]. However, lifetime extension is predicted to become economically feasible in many locations after 2020, due to design improvements and the availability of new technology [33].

#### 2.3.2 Reuse, remanufacturing and refurbishing

Component reuse involves using components/products after their designated service span for the same purpose again [51], and typically involves “checking, cleaning, repairing, refurbishing, whole items or spare parts” [[52] p.307]. In the wind industry, this can be reusing old turbines from sites that are being repowered in other locations. Some research has found that used turbines are sold in second-hand markets [53], but overall, the literature on reuse in the wind industry is scarce.

Remanufacturing is the process of restoring a used product to meet performance criteria set out by the OEM. The remanufacturing of wind turbines is now dominated by independent operators [54]. OEMs are expected to play a significant role in the future of remanufacturing as they participate in EPR [54] by providing support in post-consumer stages [6,23,32,41], such as recovery and remanufacturing of the product/component. Remanufacturing of a component/product will also improve the environmental performance of the product/component, thereby providing additional design for environment (DFE) benefits [54,55].

A remanufacturing approach can lead to a significant material reduction, increase in resource efficiency and employment opportunities, and some therefore argue that remanufacturing is the most efficient CE approach for slowing resource loops [56]. It also brings economic incentives; a recent study found the principal motivation for remanufacturing to be profitability and related market protection measures [57]. However, it was also found that remanufacturing does not consider the immobile nature of large-scale products such as wind turbines [56].

Refurbishing can be defined by similar principles. The key difference between remanufacturing and refurbishing is that refurbishing involves maintaining the overall structure of components, and sub-components are either repaired or replaced, resulting in a comprehensive upgrade [58]. In the wind industry, refurbishment is currently considered for wind turbine bearings, generators, and gearboxes [59]. However, the academic literature on refurbishing is limited.

#### 2.3.3 Repurposing

Repurposing involves using wind farm components for new applications outside the industry. There are a variety of examples, such as using turbine blades for manufacturing bridges, furniture and playground equipment, using foundations and seabed structures for artificial reefs, and more [11,[15], [16], [17], [18], [19]].

According to Joustra et al. (2021), current blade design and EOL practices seldom take repurposing into account, despite its demonstrated potential. They find the shape and composition of the material of wind turbines to have a great influence on repurposing applications, and, therefore, recommend taking subsequent cycle usage into account in the design of wind farm components [19]. Repurposing's waste management potential was also found to be affected by wind turbine blade composition, lack of awareness about the composition of materials, limited businesses with repurposing capacities, challenges to meet the waste volumes, and related costs of return logistics [27,54].

#### 2.3.4 Modularisation

Modularisation can be used to support other CE strategies, such as lifetime extension, reuse, repurposing, remanufacturing and refurbishing, by designing modular components that are easy to disassemble, clean, repair and replace [12]. Modularisation is identified as a circular strategy on its own by Velenturf (2021), and advanced planning in the design of wind turbine foundations with retrofit capabilities that are able to survive multiple lifespans are discussed in the literature [18,31]. The modularisation approach can also be used in strategy development to conduct installation and preventive maintenance, to improve the planning and execution of operation & maintenance (O&M) tasks and decommissioning, thereby saving both resources and time [60]. However, the current research on modularisation is too limited to document the effects [60].

#### 2.3.5 Repowering

Repowering is categorised into two types. Full repowering involves replacing existing wind farm infrastructure with newer and more powerful wind turbines and components, thereby resulting in an overall upgrade of the wind farm. Partial repowering usually involves replacing existing wind turbines with newer turbines, while components such as foundations and towers are reused. In both partial and full repowering, subsea cables might be reused [12,50]. Repowering alone is not sufficient to increase the share of wind energy in the energy mix [61], so newer wind farms will still need to be commissioned. However, repowering is more cost-effective than commissioning newer wind farms, so it should always be considered before decommissioning existing wind farms [20,31]. In the future, repowering will be highly dependent on decommissioning and installation strategies [62]. Repowering is placed higher than recycling in the hierarchy (Fig. 2) due to the use of existing wind farm components in the case of partial repowering.

Repowering is already a mature strategy in the onshore wind industry. The actual potential of repowering will differ significantly from the theoretical potential, as it is influenced by not only physical but also societal factors. Nonetheless, repowering offers two benefits to communities – an increase in energy supply and fewer wind turbines [63]. However, it can still result in visual disturbances due to an increase in the size of wind turbines [63]. The latter drawback is applicable to onshore and nearshore offshore wind turbines, although it would not affect the visual landscape if the wind turbines are located far away from shore.

Electricity rates and feed-in tariffs have a positive impact on the economic viability of repowering [33]. Repowering encourages investors for funding due to its capital requirement and reduced construction time. By using existing foundations and thereby avoiding seabed disturbance and pollution (caused by the installation of new foundations), repowering can provide micro- and meso-scale environmental benefits to the acclimated marine life [64]. There is a need for future research in repowering offshore wind farms, particularly in assessing the cost of wind turbines and foundations, evaluating O&M strategies and environmental impacts, and investigating the regulatory challenges [64].

#### 2.3.6 Recycling

Recycling is the circular strategy that receives the most attention in the OW industry literature, and is often used as a key metric to represent circularity in the wind industry [48,65]. Usually, recycling involves the utilisation of material obtained from wind farm components at EOL to make products with lower structural and mechanical capabilities than the original component material, which is also known as ‘downcycling’ [66].

There are a variety of recycling technologies that are used to process different parts of the wind turbine. **Fluidised bed** or **gasification** utilises air as a fluidising gas in a fluidised bed reactor to dissolve the composite matrix via a high-temperature airflow, which allows the heat generated during the recycling process to be fully utilised and obtain fibres [67]. **Solvolysis** usually involves the chemical recycling of fibres from resins using solvents [68]. **High-voltage pulse fragmentation (HVPF)** involves creating pressure waves using an electric current to disintegrate materials in water [69]. **Pyrolysis,** on the other hand, involves the decomposition of materials using high-temperature processes in an inert atmosphere [69]. **Mechanical grinding** or **shredding** involves using machines to grind or shred the fibres into granules or powders [69]. **Cement co-processing** involves separating the organic and inorganic content of fibres. The organic part, which consists of resin, core materials, and adhesive, is used as fuel, whereas the inorganic part (i.e., glass fibres) is used as raw material for the production of cement [[48], [65]]. **Solid-state chlorination** involves demagnetising permanent magnets, grounding them to powder, and then using chemical treatment to recover rare-earth elements (REE) [70]. Fluidised bed, solvolysis, HVPF, pyrolysis, mechanical grinding and cement co-processing are predominantly used to process wind turbine blades [48,65,69], whereas, pyrolysis, solvolysis, standardised metal recovery methods and solid-state chlorination are used to process REE metals, copper and steel [9,70].

Due to the presence of a variety of technical solutions, the recycling potential of wind farm components at EOL is found to be substantial, with the claimed turbine recycling rate reaching up to 85–90 % [17,48,65].

## 3 Methods

**Fig. 3:**
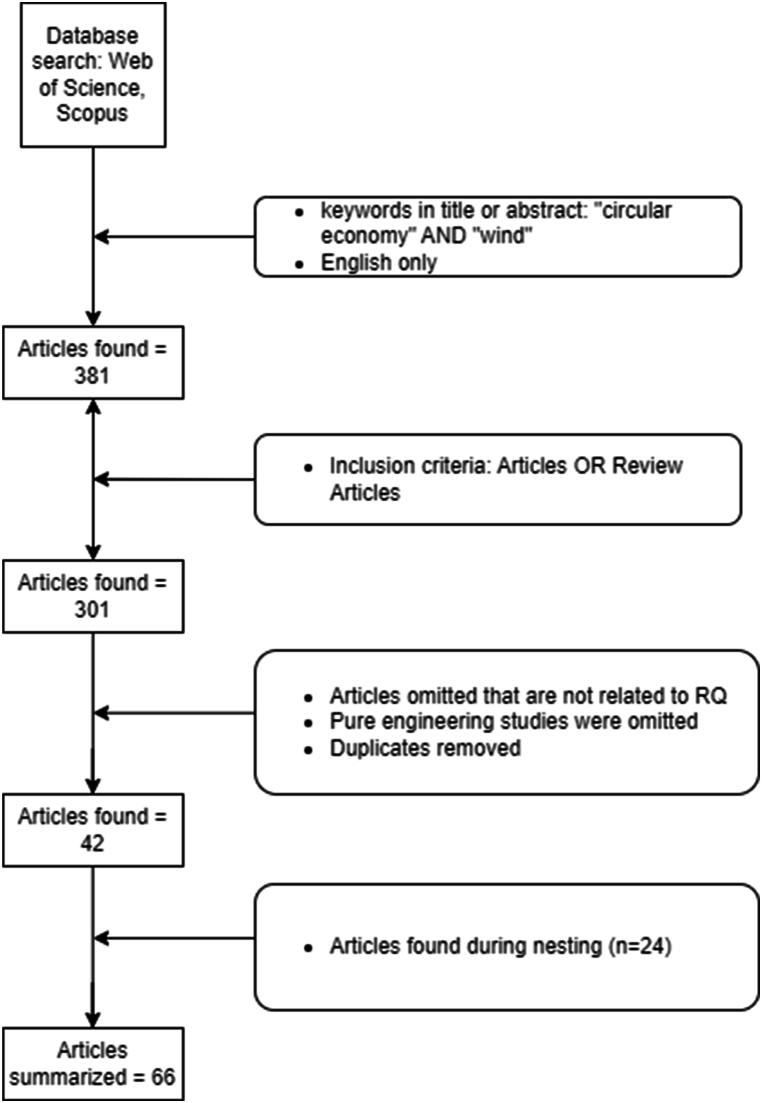
Process for identification of literature

To answer the RQ – what is the state-of-the-art in availability, heterogeneity and development of circular economy strategies in the OW industry– a systematic literature review is appropriate. Methodology of systematic literature reviews encompasses the act of assembling, arranging and assessing the current state of existing literature, where assembling refers to the identification and acquisition of appropriate literature by shortlisting the research domain, defining RQ, literature source type (e.g. journals), search mechanism (e.g. Scopus, Web of Science etc.), keyword search etc.; arranging includes organisation and purification of literature using decisions, theory, inclusion and exclusion criteria etc.; and assessing consists of evaluation and reporting of articles by using analysis and reporting methods such as thematic and descriptive analysis [71]. This review falls under the category of domain-based research, where the focus is to develop an unexplored or under-researched area/topic [71]. The process of identifying relevant studies is illustrated in Fig. 3, and the individual steps are described below.

### 3.1 Systematic literature search

To find relevant articles that are related to the proposed RQ, two databases were used: Web of Science and Scopus. The following search string was used for Scopus [TITLE-ABS-KEY ("circular economy" AND "wind") AND PUBYEAR >2000 AND PUBYEAR <2024 AND (LIMIT-TO (LANGUAGE, "English")], and for Web of Science [ "circular economy" AND "wind" (Title) or "circular economy" AND "wind" (Abstract)] with English articles only and publication date until December 31, 2023. The keywords used were "circular economy" AND ″wind'', with the assumption that CE strategies that are applicable for onshore wind should be transferable to offshore wind. The keywords were chosen to ensure a focused and relevant literature review aimed at identifying the reasons behind the heterogeneity in the development of CE strategies and systemic changes necessary to improve CE adoption in the OW industry. We acknowledge various imperatives and terms relating to CE, such as recycling and recovery, involving them in search criteria instead of “circular economy”, resulting in studies with modelling, engineering or material science-related focus. Hence, doing so would deviate from our research focus to understand the availability and commercial development of CE strategies. The term “circular economy” is often universally used in studies with a commercial resource management focus.

The literature search resulted in a total of 381 articles. The following inclusion criterion was applied: Articles OR Review Articles. Preprints were omitted as they are mentioned as non-peer-reviewed work in Scopus search. Overall, this resulted in the omission of 80 articles.

### 3.2 Choice of relevant articles

Three criteria were used to narrow down the sample to studies with specific contributions to the current research question. First, studies where the actual empirical investigation was not relevant, were removed. Subsequently, studies with purely engineering/simulation-based findings and duplicates were removed. This resulted in 42 articles that were subjected to deeper analysis.

### 3.3 Nesting

Paul et al. (2021) argue that it is challenging to conduct a literature review in a field that is not mature [71]. In this case, it is challenging both because the field is too immature to have established a common vocabulary and because the relevant research spans multiple disciplines that might use different words for phenomena that are conceptually similar. To limit this problem, we nested citations from 21 central studies, which resulted in an additional 24 studies. These articles were not identified in the keyword search because they do not use the term "circular economy", but rather the CE strategies mentioned in Fig. 2.

### 3.4 Analysis of articles

To address the RQ, both descriptive and thematic analyses [72] were performed for the identified literature. In the descriptive analysis, articles were analysed by their RQ(s), methods, year of publication, journal and relevant CE strategies. The thematic analysis focused on categorising key findings related to specific CE strategies, and rating them according to the framework in Table 1^1^ In the thematic analysis, CE strategies are categorised into two groups – namely, by assessment of higher-level CE strategies that are underdeveloped, as mentioned above in Section 2.2, and by assessment of recycling technologies. The reason for this categorisation is that recycling is the most outspread circular strategy, and therefore also contains the most detailed analysis (see Table 4).

**Table 1:**
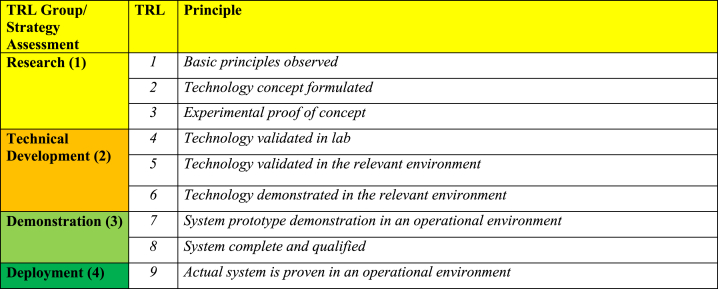
Technolgy readiness level (TRL) group/strategy assessment level framework [75]. Here, TRL is categorised into four different groups represented by four different colour codes and numbers.

TRL is a commonly used metric to assess the development stage of technology [73]. It is frequently used in the OW industry to assess the development of refurbishing, remanufacturing, repurposing, recycling, incineration (energy recovery) and landfill solutions [25,48,74]. As the exact categorisation of CE strategy based on information in the studies is challenging, this study uses four groups that are directly deducted from the TRL framework - Table 1 [75]. TRL 9 has its own group, as it is where a technology is actually considered technically and commercially viable [73].

Although TRL is intended to assess technology development, we argue that the proposed TRL group framework is transferrable to measure the maturity of remaining CE strategies – lifetime extension, reuse, modularisation and repowering (Fig. 2) – as the development of these strategies also undergoes the same phase of development as TRL group level. However, in this case, it is denoted as ‘strategy assessment level’. Therefore, by applying the TRL group/strategy assessment level, we proceeded to code the invested CE strategies in each study according to this framework.

**Fig. 4:**
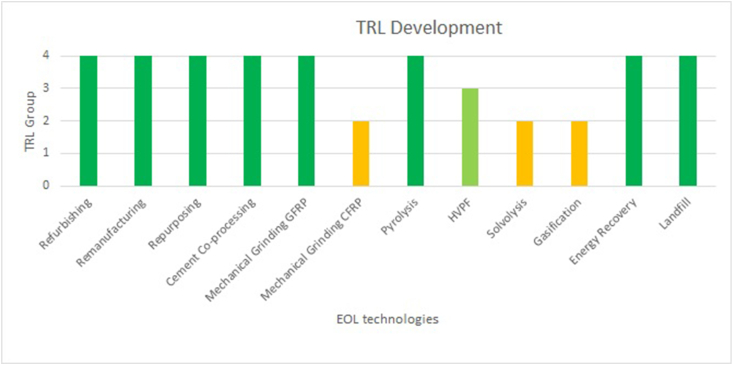
Technolgy readiness level (TRL) group level development of end-of-life (EOL) strategies (adapted from Refs. [25,34,48]). Here, GFRP – glass fibre-reinforced polymer, CFRP – carbon fibre-reinforced polymer and HVPF – high-voltage pulse fragmentation.

By applying the abovementioned framework and using the TRL value obtained from peer-reviewed studies and the wind industry, the following reference values were obtained (Fig. 4). These values will later be used as a baseline to compare the findings.

## 4 Results

The 66 identified studies were analysed using descriptive and thematic analyses. We will now proceed with a descriptive presentation of the studies before describing the analyses according to the TRL group/strategy assessment levels in Table 1.

### 4.1 Description of articles in the literature review

The sample shows a broad mix of applied research methodologies. Most of the studies adopted a qualitative approach, followed by quantitative approaches, techno-commercial analyses, mixed methods and literature reviews, respectively (see Table 2). The high number of qualitative and quantitative studies is an indication of the advancing nature of research on CE in the wind industry. However, the variety of methods also suggests that research has reached a certain level of maturity, where different approaches are used to investigate the phenomenon.

**Table 2:** Methodology employed in selected articles.

Qualitative	Quantitative	Literature Review	Mixed Methods	Techno-commercial
20	16	8	11	11

The broad variety of research methods is a good foundation for knowledge accumulation. However, we also observe a high level of fragmentation in terms of publication channels. The 66 studies were published in 35 different journals over a 12-year period. The number of studies is rapidly increasing, as 35 of the 66 studies were published between 2021 and 2023. This can be seen as an indication of the positive push from the research community towards the CE transition in the OW industry. Moreover, the fragmentation of publishing channels and significant recent additions to our knowledge base comprise the primary motivation for this literature review.

Another factor that contributes to fragmentation is the observation that the studies consider the circular strategies for both whole wind farms as well as a variety of different components of the wind farm. Most studies consider CE strategies for whole wind farms and are predominantly targeted at the firm level. Regarding components, circular strategies for wind turbine blades dominate and receive much more attention than other central parts of wind turbines, such as generators/rare earth elements (REE), foundations and scour protection. This is justified as wind turbine blades are often regarded as the most challenging part of the wind turbine to recycle [6,9,11,17,25,32,48,65,76,77]. By 2050, it is estimated that the global recycling demand for composite waste from wind turbine blades will reach 42 million tonnes per year [25].

### 4.2 Assessment of research on circular economy strategies in the offshore wind industry

**Fig. 5:**
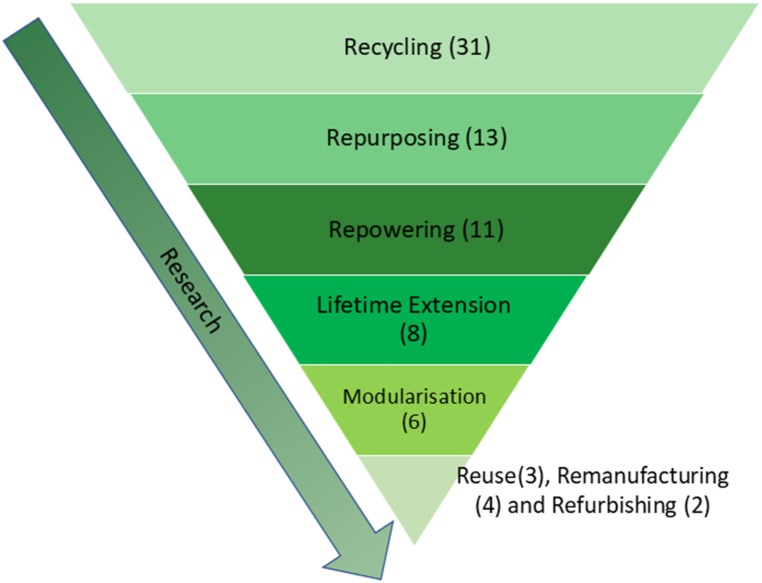
Research on circular economy strategies in the offshore wind industry

The second part of the analysis assesses research on CE strategies. As mentioned above, we observe great heterogeneity in the research. Recycling is the most discussed strategy (31 studies). Research on other CE strategies was highly variable, with only two studies on the refurbishing approach (Fig. 5). What is interesting here is that the behaviour observed in Fig. 5 is contrary to that observed in Fig. 2 – ie., CE strategies with higher sustainability potential are the least researched.

#### 4.2.1 Assessment of higher-level circular economy strategies

Table 3 shows the assessment of investigated CE strategies according to the framework in Table 1. It excludes recycling strategies as there is a more extensive list of recycling strategies, which allows for more fine-grained analyses. Studies are presented in order of publication date to illustrate the development of technological and market maturity.

**Table 3:**
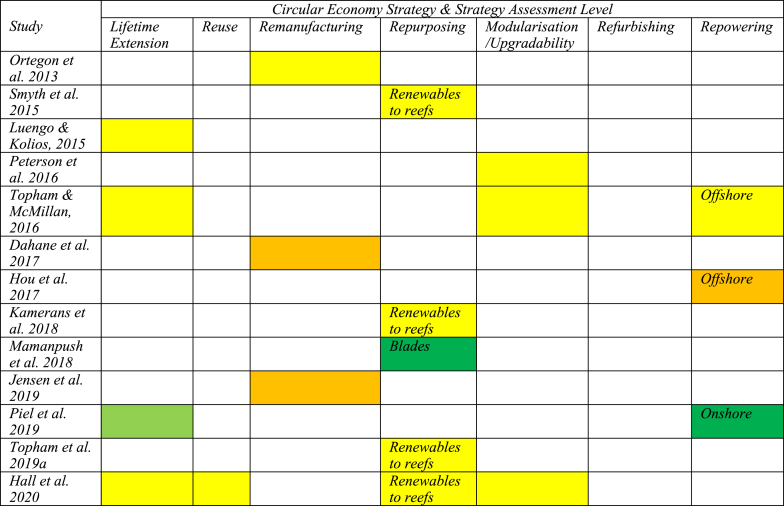

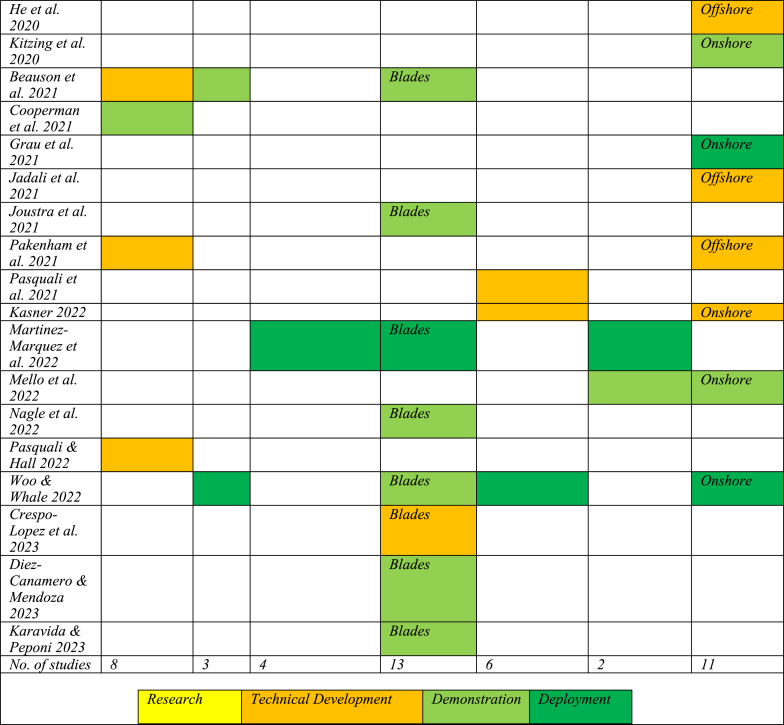
Assessment of circular economy strategies using strategy assessment level.

The results show a clear overall development of CE strategies in OW towards more technological and market maturity. All studies published before 2017 are classified as research (TRL 1–3: TRL group 1), and except for one, all studies published in 2020 or later are at later stages of technical development, demonstration, or deployment.

Apart from the expected development pattern, the analysis also showed variation in maturity across CE strategies and across different studies. For instance, the development of lifetime extension was found to be different in different articles, and it does not seem to follow a strict chronological development pattern. Analysis conducted by Hall et al. (2020), Beauson et al. (2021) and Cooperman et al. (2021), which were written at almost the same time, resulted in significantly different results. Hall et al.’s (2020) study, which was conducted based on an environmental impact assessment of 12 onshore and offshore wind farms in the UK, suggests that lifetime extension was at the research stage, according to the framework suggested in Table 1 [18]; Beauson et al.’s (2021) findings indicate that lifetime extension was at the technical development phase [27]; and Cooperman et al.’s (2021) study, which was based on quantitative waste material data in the USA, suggest that lifetime extension was already at the demonstration stage [36]. A similar variation in strategy assessment level was found in the development of other CE strategies – repowering, reuse, refurbishing, repurposing, and modularisation.

Interesting observations also emerge when we look across CE strategies. First, there is heterogeneity in the amount of attention that the different CE strategies receive from the researchers. In addition to recycling (31), the most studied strategies are repurposing (13) and repowering (11). These are also the CE strategies that are found to be the most mature CE strategies after recycling.

The strategy of repurposing is dependent on factors such as damage to blades, demand for blade models, blade model dimensions and the accurate estimation of blade waste [27]. Repurposing falls into two categories – renewable to reefs and repurposing of blades. In the renewable to reefs scenario, seabed structures such as foundations and scour protection are left in place after decommissioning to support marine habitats [11,15,16,18]. The potential of this method is not yet proven, and it is believed to be highly site-specific and species-specific [15,18]. The potential of this repurposing scenario was found to be at the research stage. In contrast, blades can be repurposed for manufacturing bridges, road barriers, furniture, playground equipment, etc. [19,27,78]. The business viability of blade repurposing is already proven, although it is believed to be affected by barriers such as the shape and composition of the material [19], limited repurposing applications and related marketplaces to sell repurposed goods [79], and unknown residual structural properties of the original material [21]. Additionally, the processing capabilities of these methods have not yet been tested, as the offshore wind farm decommissioning peak has not yet been reached [27,80]. It was found that the repurposing of blades is in the late demonstration/early deployment phase, but that this currently represents a limited area of business.

The modularisation strategy has the potential to facilitate higher-level CE strategies, as shown in Fig. 2. Modularisation could be used in the design of foundations with retrofit capabilities that can survive multiple lifecycles, and could also be used for partial repowering [18,31], to reduce wind farm maintenance time, and to improve planning and execution of O&M activities [60]. Siemens Gamesa's Energy Thrust [81] and Vestas' Power Plus [82] are offering upgrade packages for wind turbine performances by increasing the wind turbines' annual energy output by up to 5 % [26]. However, in all the other identified studies, modularisation was found to be at the research or technical development phase.

Repowering, on the other hand, is considered a mature or near-mature technology for onshore wind [26,33,61,63,83], apart from one exception where it was found to be at the technical development phase, as the results were tested in a simulation for repowering after a 50-year wind farm lifecycle [84]. The remaining five studies on repowering strategies concerned offshore wind farms where repowering was observed in the late research or early technical development phases, and had a way to go before it would become a viable market option. Offshore wind farm repowering was found to be affected by several factors, such as installation and decommissioning technology [62], the environmental impact of repowered turbines [64], cost comparisons between decommissioning and repowering [64], limited literature availability on EOL scenarios, and the scarcity of data on lifetime extension and decommissioning processes [85].

#### 4.2.2 Assessment of recycling technologies

Despite its low sustainability potential, recycling was found to be the most widely researched CE strategy (Fig. 5). Recycling is used as the principal indicator to represent the circularity of OW businesses [48,65]. This is surprising, given that recycling is the least sustainable CE strategy, as shown in Fig. 2. The studies consider a variety of recycling technologies (see Table 4), and are rated according to the TRL group framework mentioned in Table 1. Compared to other CE strategies, the overall development of recycling technologies was found to be more mature, as indicated by the presence of more green areas in Table 4.

**Table 4:**
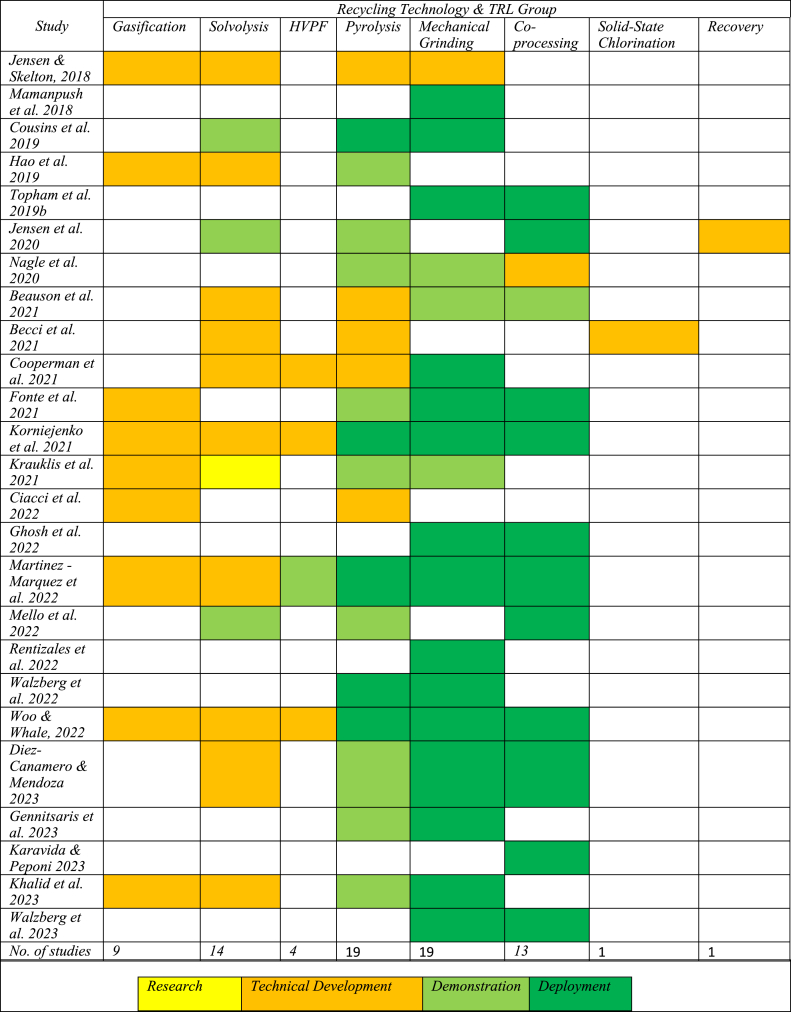
Assessment of recycling technologies using technology readiness level (TRL) group framework.

The overall trend of increasing maturity is less clear from the recycling strategies. This can be explained by the fact that the studies were published within a shorter period (2018–2023) and that some studies had already reported the stage of deployment in 2018. However, there is still considerable heterogeneity across recycling strategies.

Mechanical grinding and co-processing of WT blades were observed to be late demonstration/near deployment technologies, which resembles the findings obtained from the industry (

Fig. 4). However, it was also found that co-processing capabilities in Germany and Ireland were at different levels [86], which is noteworthy. With just one exception, all the articles published in 2022 and 2023 reported pyrolysis at the late demonstration/near deployment phase. Here, a new process of pyro-gasification was tested to recover carbon fibres at pilot scale [39]. Gasification, HVPF and Solvolysis, with slight variations in their development, were almost found to be at the technical development stages. A study conducted in 2022 found both solvolysis and gasification to be at TRL 5–6 (late technical development), and HVPF at TRL 8 (late demonstration phase) [25]. Despite strongly established recovery solutions, there is uncertainty about the actual recovery rate [9].

## 5 Discussion

If we summarise the key findings from the literature on CE strategies associated with the OW industry, we find that it is scattered across a broad range of journals, it uses a variety of research methods and units of analysis, and there is a significant number of recent contributions. The nature of this research, therefore, requires periodic reviews to establish the current knowledge base. Regarding the CE strategies, we observe that most studies focus on recycling strategies (31 of a total of 66). Recycling constitutes the benchmark for the circularity of the industry, even though it scores relatively low on overall sustainability. These studies are all relatively recent (all published in 2018 or later) and show that recycling is a mature strategy, both in terms of technology and market availability, but the maturity varies according to geographical location. Other higher-level CE strategies are found to be less mature, and there are many studies that are exploratory in nature. There is, however, a clear pattern towards higher levels of technological and market maturity. Among the higher-level CE strategies, we observe heterogeneity in the development, but there is also a general pattern that strategies with less potential for sustainability (Fig. 2) are the most developed. We will now proceed to discuss the underlying factors that help us understand this variation.

**Fig. 6:**
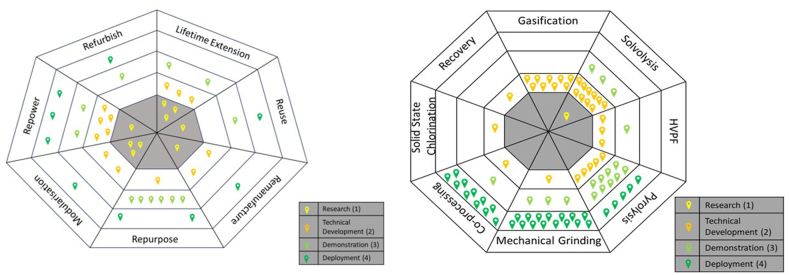
Variation in the development of higher-level circular economy strategies (left) and recycling technologies (right)

Fig. 6 provides a visual representation of the variation in the maturity of CE strategies associated with the OW industry. As previously mentioned, the observations are that CE strategies high in the waste management hierarchy (Fig. 2) are less developed than those at lower levels, such as recycling, and there is significant variation in the maturity levels reported from different studies. These observations deserve further discussion, and the reviewed studies point to several alternative explanations.

The studies point to the following factors to understand the heterogeneity in the maturity of CE strategies.

1. There are site- and country-specific developments of CE strategies [20,27,[31], [32], [33],^41^]. Heterogeneity in the industrial context and the availability of relevant knowledge can therefore cause heterogeneity in suitable strategic responses.
2. Another important source of heterogeneity lies in constraints and incentives in regional regulations. In other words, variations in standards and regulations related to recycling activities, component waste, landfill bans and similar can provide different motivations and possibilities for CE strategies [27,34].
3. Similarly, there can be heterogeneity in alternative costs for CE practices. For example, for many actors, it might be cheaper with landfill or energy recovery solutions compared to CE strategies [[34], [35], [36],^48^].
4. Another explanation could derive from the existence of the global waste trade from high-income countries to low-income countries with insufficient waste management capabilities, less rigorous health and environmental policies, and thus comparatively cheaper waste processing costs [87].
5. Other studies point to information challenges. One information challenge is that there is limited available and reliable information on the environmental footprints of waste management, in particular, related to CO_2_ emissions of transportation and the actual efficiency of waste processing by CE strategies, i.e. how much of the segregated waste is processed using CE strategies [87].
6. Some other studies point to a lack of available and reliable information on environmental impacts and CO_2_ emissions caused by virgin material extraction and production [88,89].
7. There is a lack of experience in decommissioning activities, especially related to regulations of waste management, planning processes and environmental impacts [9,11].
8. Finally, some studies point to a coordination problem where a lack of coordinated collaboration between industrial actors such as material suppliers, wind turbine component manufacturers, wind farm owners, and operators halts the transition to CE practices [32,57].

All of the above arguments are credible explanations based on the economic rationality of the actors. In other words, actors adopt CE strategies according to economic interests and the opportunities that lie in the context in which they operate. However, they fail to explain the observation that lower-level CE strategies are more developed than more sustainable higher-level CE strategies, as defined in Fig. 2.

The recycling strategy is found to be more mature than the other CE strategies, and it serves as the principal indicator for the circularity of the wind industry, with claimed wind turbine recycling rates reaching up to 85–90 % [17,48,65]. High levels of maturity and high levels of theoretical recycling rates make sense, as recycling solutions are often transferable across industries. However, there are several indications that the actual recycling rates are lower. The argument stems from the following observations.

1. OW value chain actors lack experience or information (or both) related to decommissioning activities, waste management capacities, material reuse, and availability of commercially viable recycling methods [9,11,12,27].
2. The cost of recycling is not yet compatible with the low cost of landfilling and energy recovery [[34], [35], [36]].
3. There can be differences in the interpretation of the term ‘recycling’. For example, for waste management companies, the recycling rate can be the proportion of waste separated for recycling relevant to the total generated waste, whereas for recycling companies, it is the proportion of waste that is actually recycled to the total waste that they received for recycling [87].
4. There is a lack of reliable and accurate information on transboundary effects – i.e., the effect of physical distance on transporting waste to recycling or waste management plants and related CO_2_ emissions [29,87,90].
5. Finally, as was also found in this study, some of the recycling strategies have not reached the level of commercial viability and suffer from degradation of materials, high process-related emissions and other undesirable environmental impacts [14,34,36,48].

In addition to these factors, this study has revealed that a general analysis of the TRL framework fails to recognise the variations in region-specific factors such as heterogeneity in industrial contexts, availability of relevant knowledge, constraints and incentives in the regional regulatory system, and heterogeneity in alternative costs. Cumulatively, these factors explain why recycling alone should not be used as a default circularity indicator and why higher-level CE strategies (Fig. 2) and regional-specific factors should be taken into account.

Moreover, several studies point to a broader system-level assessment of factors than recyclability to evaluate the sustainability level of CE strategies in the wind industry. They argue that fundamental changes are required in the way in which CE strategies are measured, and point to the need to include an assessment of the environmental footprint of material extraction [88], how much materials are deployed and how they flow across value chains [9], the amount of waste material generated, the environmental efficiency of local waste management and processing facilities, and the environmental footprint of logistics [87].

Current research indicates that to succeed with CE strategies and resolve the material challenges of the OW industry, it will require the activation of resources across value chains as well as system-level changes. The successful implementation of CE strategies in the OW industry will require support from adjacent sectors – for example, the development of remanufacturing will depend on OEMs or similar specialist businesses, whereas the development of recycling will be dependent on wind OEMs, wind farm owners/operators, recycling and waste management businesses, recycling locations and facilities, as well as resource demand of secondary manufacturing sectors [12]. This collaborative effort will also aid in improving the business case for CE strategies in the OW industry, growing and developing local supply chains, and reducing the risk for the OW industry related to the sourcing of materials and market opportunities for circularly processed goods [12].

The latter observation that successful implementation of CE strategies in the OW industry requires the activation of resources from multiple stakeholders across value chains represents a major strategic challenge for innovative firms that seek to implement CE. As a recent study showed, using game theory, the successful implementation of CE strategies requires a simultaneous, coordinated and dedicated adoption of CE practices from a sufficient number of actors to form a complete circular value chain [91]. If not, first-moving circular innovators will systematically stand at a strategic disadvantage to incumbents that remain in the old linear system. This is labelled ‘the curse of the circular innovator’, and will remain a significant strategic challenge even though CE strategies (as discussed in this study) are technologically and market mature. This perspective can also help us to understand the limited adoption of CE strategies in the OW industry and elsewhere.

## 6 Conclusion

The OW industry is expected to grow significantly over the next few decades. Moreover, there will be a steep growth in decommissioning activities by the end of this decade, as a significant number of first-generation wind turbines are approaching the end of their expected lifespans. In order to not turn a sustainable and renewable solution to the climate challenge into a resource challenge, there is a need to develop a circular OW sector that is economically, socially and environmentally sustainable. However, research has found that a transition from a traditional linear economy to a circular economy is challenging for several reasons. The contribution of this study is the review of the technological and market maturity of the current and emerging CE strategies for the OW industry, and the discussion of the reasons for the variation in development.

The study finds that, in the context of the OW industry, the academic literature on CE is highly fragmented across a high number of journals and across a wide area of topics. We also find it to be limited. However, we have also observed a substantial increase in CE research in OW over the past few years, and there is, in general, an increasing trend for technological and market maturity.

This study observed heterogeneity across different CE strategies, where some are more technologically and market-mature than others. This is not surprising, but what is more intriguing is the clear trend that the CE strategies with the lowest potential for sustainability in the waste management hierarchy receive the most attention from researchers and the industry, and show the highest levels of maturity. This is particularly expressed by the high number and maturity levels of recycling studies. The reviewed studies offer several credible arguments for why we observe heterogeneity across CE strategies, but we lack knowledge on why the CE strategies with the highest levels of potential for sustainability receive less attention and report low levels of technological and market maturity. We call for more research to address this question and focus more development efforts towards high-level CE strategies.

Future studies could also look at other emerging circular strategies, such as material and component substitution, particularly in the design of REE-free generators. Currently, China has dominance over the REE supply chain [[92], [93], [94], [95]], which could be affected by geopolitical restrictions and supply chain shortages [93]. There are promising breakthroughs in the substitution and recycling of REEs in generators [92,96], but these are not considered to become viable options in the foreseeable future [9,96,97]. Future studies could also look at learnings related to EOL management from more mature markets. For example, countries such as Canada and the US, where offshore wind is emerging industry, could learn valuable lessons from the EOL management of matured European offshore markets such as Denmark [26].

A separate analysis of the recycling studies has also shown heterogeneity in maturity levels across different recycling strategies, but in general, the maturity levels related to recycling are higher than for other CE strategies. This is an important observation, as recyclability forms the baseline metric for the circularity of the OW industry. Some studies and industrial reports have claimed a wind turbine recycling rate as high as 85–90 %, but the findings of our study suggest the actual rates might be significantly lower than this. Some of the reasons for this are industry-specific, such as low levels of recycling experience, confusion of terms and lack of information. Other reasons are more general, such as lower costs of landfill or energy recovery compared to their circular counterparts, and high levels of material degradation associated with some of the matured recycling technologies. However, we also argue that some of the problems arise from the use of TRL metrics to establish a theoretical level of recyclability in the industry. TRL does not account for the country- and region-specific variance in technology development, or the heterogeneity in policies, regulations, and the availability of knowledge or related industries.

The study concludes that, in order to develop higher-level CE strategies for the OW industry, we need system-level change that includes fundamental changes in the way we measure circularity, early consideration of EOL strategies in the planning phase of the wind farm, and the design of components with their EOL strategy in mind. Also, collaboration across the value chain involving a range of actors who are part of wind farm planning, commissioning and decommissioning, and cross-border trade-off of information with other relevant sectors needs to be boosted. This will help to ensure the coherent, simultaneous, multi-faceted and dynamic growth of CE strategies.

A potential limitation of this review is that the findings related to heterogeneity and the development of CE strategies are predominantly based on peer-reviewed studies. Future studies could include empirical ways of validating this data by carrying out roundtable discussions and interviews with industry practitioners, researchers and policymakers, as such data could accelerate the transition of CE in the OW industry.

## CRediT authorship contribution statement

**Pankaj Ravindra Gode:** Writing – original draft, Visualization, Methodology, Formal analysis, Conceptualization. **Arild Aspelund:** Writing – review & editing, Supervision, Conceptualization.

## Ethics declarations

Review and/or approval by an ethics committee was not needed for this study because it is based on peer-reviewed work.

Informed consent was not required for this study because it is based on peer-reviewed work.

## Data and code availability

Data will be made available on request.

## Declaration of competing interest

The authors declare the following financial interests/personal relationships which may be considered as potential competing interests: Pankaj Ravindra Gode reports financial support was provided by Norwegian Research Centre on Wind Energy (FME NorthWind).

## Footnotes

1 Here, it important to note that 12 of the 66 articles selected resulted in inconclusive results after applying the framework suggested in Table 1. However, these articles are included in the analysis for their contribution related to the RQ.
